# Amending the literature through version control

**DOI:** 10.1098/rsbl.2022.0463

**Published:** 2023-01-18

**Authors:** Adam Kane, Bawan Amin

**Affiliations:** ^1^ School of Biology and Environmental Science, O'Brien Science Centre, University College Dublin, Belfield, Dublin 4, Ireland; ^2^ Faculty of Social and Behavioural Sciences, Utrecht University, Utrecht, The Netherlands

**Keywords:** corrections, open science, reproducibility, research practices, transparency

## Abstract

The ideal of self-correction in science is not well served by the current culture and system surrounding amendments to published literature. Here we describe our view of how amendments could and should work by drawing on the idea of an author-led version control system. We report a survey (*n* = 132) that highlights academics' dissatisfaction with the status quo and their support for such an alternative approach. Authors would include a link in their published manuscripts to an updatable website (e.g. a GitHub repository) that could be disseminated in the event of any amendment. Such a system is already in place for computer code and requires nothing but buy-in from the scientific community—a community that is already evolving towards open science frameworks. This would remove a number of frictions that discourage amendments leading to an improved scientific literature and a healthier academic climate.

## 1. The problem

We, and many other academics, argue that the current model of scientific publication hinders the capacity for self-correction [1–3]. People make mistakes and the peer review system cannot catch them all [4–6]. Yet the number of steps involved in correcting previously published research acts to discourage what should be a straightforward process that is in the hands of the original authors [1,2].

This is to say nothing about academia's difficult relationship with authors owning mistakes [1,2,7]. Indeed, a recent survey explored the ‘emotional, reputational and practical’ barriers standing in the way of correction [8]. The same article showed that a significant proportion of those surveyed would not report a major mistake in a high-impact paper that affected the paper's conclusions. Certainly, nobody likes to be wrong and the mental anguish of people who go through correcting or retracting something they spent years of work on is palpable [9,10]. However, that some commentators deem these acts as heroic shows just how far away we have moved from the self-correcting ideal—heroes are exceptional, mistakes are not [11].

Then there is the simple opportunity cost of dealing with relatively minor issues in a paper. Those that vex but are not worth the time and effort to correct [8], or instances where you have a new dataset that could supplement previous work but is not worth publishing separately.

This is all aggravating because the Internet offers us a way forward through version control. The static face of academic publications is a holdover from times where print held sway, but the dynamic and reflective nature of the Internet is much more in keeping with how science should operate [12]. We propose that researchers should create versions of their own papers that record amendments to their work while retaining the original published manuscript for reference. Here we report results from our own survey to assess the level of support for our proposal, detail how the system would work, including its benefits, and respond to potential counterarguments.

## 2. Survey

We developed a survey to enquire about scientists' knowledge, exposure and experience with the process of issuing a correction (see the data in the electronic supplementary material [13]). We also asked the following statement that relates to our proposed solution:

*Imagine adding a link to published papers, which would direct readers to an online, open and updateable repository (*e.g. *GitHub, OSF, etc.). Such a framework would be used by the authors of the paper to add any update to said paper. Updates can be amendments, text corrections, additional data and analysis. These updates would not alter the journal's version of record. This system would not need any involvement from the journals (except providing the link).*

We then finished with a suggestions box so that people could expand on this or related points. We built the survey using Google forms and disseminated it over Twitter as well as contacting 13 researchers who we know had an interest in this area with the request to spread it through their Twitter network.

## 3. Results

We recorded 132 respondents (see the supplementary data for full results), two-thirds of whom were in the life sciences across career stages. Remarkably, almost a third of respondents were unaware it was possible to make amendments to peer-reviewed publications. In keeping with previous results [8], the percentage of people who have considered amending their work exceeded the percentage of those who have attempted an amendment. Out of the 19 people who successfully amended their work, only a quarter (five) agreed that the current system worked well. The remainder were either neutral (eight) or disagreed (six). There were a variety of reasons given by the 34 people who had not formalized an attempt for amendment including lack of clarity as to how to proceed (20), hassle with the publishers (12), the time needed (nine), embarrassment (eight) and scorn of their peers (three). In response to our proposed solution only around 7% (nine) of people disagreed with the idea, the rest were in support (approx. 61%, 81 people) or conditionally so (approx. 32%, 42 people). We note that these results are biased in focusing on Twitter users and people engaged in Open Science discussions.

## 4. Proposal

We suggest that every published paper includes a link to a page controlled by the authors, for instance, a GitHub repository (or equivalent, e.g. Open Science Framework). This is already in place for things like supplementary data and code in line with open science practices [12,14]. Here, the authors could detail errors, amendments and additional analyses using the ‘readme’. This readme would include extensive information on the differences between the versions. We note that these need not be reserved for coding issues, everything from a fundamental mathematical error to an awkward sentence could be fixed. Because of the version control features inherent to Git, the original, peer-reviewed version would still be accessible. Further, many researchers already use GitHub to record new versions of R packages commonly used by scientists across many fields (e.g. the tidyverse – https://github.com/tidyverse/tidyverse/releases).

**Figure 1.**
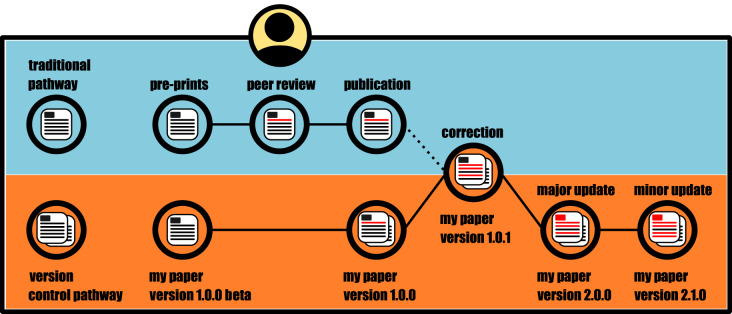
Graphical representation of our proposed system that shows its relationship with the current system. Our system deals with the barriers (dashed line) that are currently restricting authors from correcting their published work.

Readers who follow a GitHub repository could get email notifications anytime a change is made. Authors could also draw attention to this new version by creating a new file on their Google Scholar profiles, ResearchGate accounts etc. Although authors can decide for themselves how to label their versions, we suggest an X, Y, Z numbering system that follows best practice for version control through semantic versioning (https://semver.org/) where X is a major additional analysis, Y is a minor additional analysis and Z is a correction. This would display as—‘Darwin, C. (1859). On the Origin of Species. - VERSION 1.0.0’ for the initial publication, and a correction as ‘Darwin, C. (1859). On the Origin of Species. - VERSION 1.0.1’ with updates containing a link to the GitHub release page (figure 1). Such an approach could also be done retrospectively for papers that have already been published and lack an internal link. Authors could create an updateable repository and advertise any amended versions post hoc. Finally, we advocate that authors create a Zenodo version with a DOI for updates which would ensure fixity of new versions of their manuscripts.

## 5. Benefits and potential issues

There are some similar initiatives which speak to the scientific dissatisfaction with the static publication model. Living Reviews have version control but at the level of a review article, or F1000research/ Evolving Papers which are editor-controlled forms of what we suggest. Then there is Manubot, which details a similar approach and specifically requires GitHub, but which mostly targets the process pre-publication [15].

The main benefit to our system is that it allows previously published work to be amended and/or updated in a straightforward way because it is author led. This takes out the middleman of the journals who do not need to be involved at any step once the paper is published in its initial, peer-reviewed state (see responses to question 9 in the supplement). The purpose of the link could be raised by the authors to the journal editors during submission.

We hope that our relatively frictionless system would engender a culture of correction including an awareness of correction as a possibility [2]. Adhering to semantic versioning would grant authors a natural flow if they use preprints by referring to this version as the beta stage. Further, it would offer plenty of grist for students to verify/update/correct the work of their supervisor—typically a principal investigator who may have more time constraints. This would stand to benefit the students who could point to tangible outputs on their CVs. It would also empower authors to raise flags about their own work even if they have not yet had time to address them (cf. the Loss-of-Confidence Project [7]).

There are some potential issues with our proposal. For one, there is a separation between the amendment and the journal, although this could also be considered as a positive as argued before. The onus here is on the authors to advertise any changes. The semantic versioning system using the same title as the paper should help here in terms of search engines turning up both. Further, as noted earlier, interested readers could follow a GitHub repository of a paper so they are alerted to any changes. Non open access journals could take issue with wholesale replication of the publication outside of their website. Here authors could discuss changes to specific sections of the paper that only those with access to the paper could make sense of. Then there are questions of what to do if the paper is so flawed that it needs to be retracted or instances of malpractice. In this case we agree that contacting the journal is best practice. Though here again, initial concerns could be flagged early by the authors.

We argue that the concerns raised in our survey as well as our own thoughts of potential shortcomings are not unique to this system. Disagreement among co-authors, visibility of the amendment and malpractice are some issues that pertain just as much to the current system of corrections [4,16]. For instance, those scientists who engage in fraud [17] are just as unlikely to flag their malpractice with our system as with the current set-up. Indeed, we note that ours is an additional system, it allows for immediate corrections, where authors can still go through the established route if they desire.

## 6. Conclusion

Our findings add weight to the view that the current system of academic publication hinders science by disincentivizing corrections and amendments to published literature [3]. A system of version control, as we detail here, would offer numerous benefits to what is an inherently dynamic and imperfect process of discovery [18], all the while keeping the version of record. Our work will also serve to highlight corrections as a possibility, something that many of our survey respondents were unaware of. The fundamentals to this system are already out there and simply require buy-in from the community. Although it is not a perfect system, we believe a constantly updating and updateable literature that is in the hands of the authors better reflects the ideal of science. With this, we can take another step towards a more robust and reliable scientific literature and an improved working academic climate.

## Data Availability

Open Science statement supplementary data can be found here: https://doi.org/10.5281/zenodo.7332268 [13] and updates here: https://github.com/kanead/Corrections.
